# Enhancement of interaction of L-929 cells with functionalized graphene *via* COOH^+^ ion implantation *vs.* chemical method

**DOI:** 10.1038/srep37112

**Published:** 2016-11-15

**Authors:** Meng-li Zhao, Xiao-qi Liu, Ye Cao, Xi-fei Li, De-jun Li, Xue-liang Sun, Han-qing Gu, Rong-xin Wan

**Affiliations:** 1 Energy & Materials Engineering Centre, College of Physics and Materials Science, Tianjin Normal University, Tianjin 300387, China; 2 Tianjin International Joint Research Centre of Surface Technology for Energy Storage Materials, Tianjin 300387, China; 3 Department of Mechanical & Materials Engineering, Western University, London, ON, Canada; 4 Tianjin Institute of Urological Surgery, Tianjin Medical University, Tianjin 300070, China

## Abstract

Low hydrophilicity of graphene is one of the major obstacles for biomaterials application. To create some hydrophilic groups on graphene is addressed this issue. Herein, COOH^+^ ion implantation modified graphene (COOH^+^/graphene) and COOH functionalized graphene were designed by physical ion implantation and chemical methods, respectively. The structure and surface properties of COOH^+^/graphene and COOH functionalized graphene were characterized by scanning electron microscopy (SEM), transmission electron microscopy (TEM), Fourier transform infrared spectroscopy (FTIR), Raman spectroscopy, X-ray photoelectron spectroscopy (XPS), and contact angle measurement. Compared with graphene, COOH^+^/graphene and COOH functionalized graphene revealed improvement of cytocompatibility, including *in vitro* cell viability and morphology. More importantly, COOH^+^/graphene exhibited better improvement effects than functionalized graphene. For instance, COOH^+^/graphene with 1 × 10^18^ ions/cm^2^ showed the best cell-viability, proliferation and stretching. This study demonstrated that ion implantation can better improve the cytocompatibility of the graphene.

In recent years, graphene, as an outstanding two-dimensional (2D) monolayer of carbon atoms, has attracted considerable attention due to its extraordinary properties including large surface area, excellent thermal conductivity, large mechanical fracture strength, high optical transmittance and superb charge-carrier mobility^1^,^2^,^3^,^4^,^5^,^6^,^7^,^8^. In the biomedical field, such as tissue engineering, implants, bioimaging, biosensors, drug delivery and regenerative medicine, the graphene with unique physicochemical properties was expected to show enhanced performance originating from aforementioned advantages^9^,^10^,^11^,^12^,^13^,^14^. However, the low hydrophilicity, insufficient biocompatibility and bioactivity of graphene prevent from its biomedical applications^15^. Therefore, the surface functionalization of graphene is a crucial process to promote the biological properties^16^,^17^.

Previous studies revealed that the COOH modified carbon nanotubes (COOH-CNTs) exhibited the promising effects on biocompatibility due to the active functional groups^18^. In comparison to CNTs, graphene with larger surface area and more active edges is more likely to form defects with easy modification^8^,^14^. Hence, tailoring the biological properties of graphene *via* carboxyl should be an ideal approach to extend the potential applications of graphene in biomaterial field^19^.

Ion implantation, which can modify the structure of a target-near-surface though high-energy ions, is one of the most powerful techniques in modifying surfaces^20^. Furthermore, ion implantation with the technological simplicity and cleanliness mainly modifies the surface characteristics without affecting bulk properties^18^,^21^. In this research, graphene were bombarded by ion implantation, and the COOH^+^/graphene with different implant doses were created by adjusting the injecting time. The surface modification *via* chemical method is also considered to enhance the biocompatibility of graphene^22^. Herein, both ion implantation and chemical method were employed to modify the graphene, as described in Fig. 1. The interaction between cells and graphene, COOH^+^/graphene or COOH functionalized graphene were evaluated by L-929 fibroblast cells *in vitro*.

## Results

### Characterization of graphene, COOH^+^/graphene and COOH functionalized graphene

**Figure 2:**
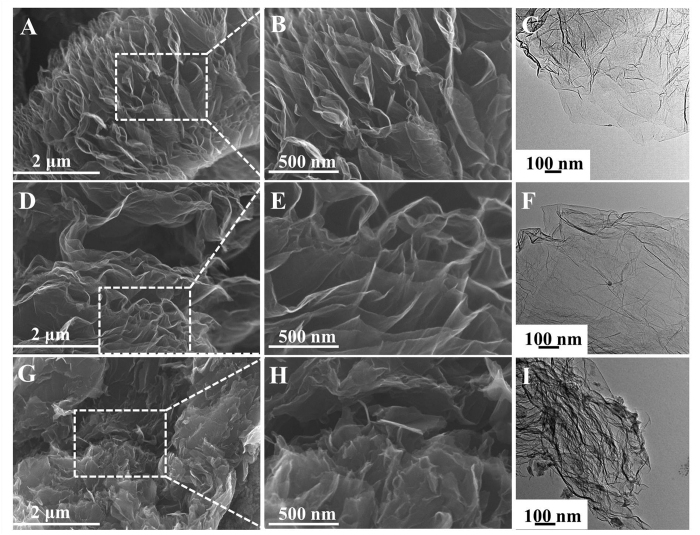
SEM images of graphene (**A**,**B**), COOH^+^/graphene (**D**,**E**), COOH functionalized graphene (**G**,**H**). TEM images of graphene (**C**), COOH^+^/graphene (**F**), COOH functionalized graphene (**I**).

SEM and TEM were used to characterize the morphologies and structures of graphene and its functionalized derivatives, as shown in Fig. 2. Graphene with the thin, randomly crumpled sheets is associated with each other, as it is showed in Fig. 2(A–C). The COOH^+^/graphene (Fig. 2D–F) exhibits some wrinkled edges and layer-by-layer structure. Both graphene and COOH^+^/graphene reveal similar microstructures, indicating that the ion implantation does not significantly affect the 3D profile of graphene. However, the obvious morphology changes were observed after chemical functionalization of graphene. SEM and TEM images (Fig. 2G–I) of COOH functionalized graphene can be clearly identified as ‘building blocks’ and reveal obvious aggregation throughout oxidization process.

**Figure 3:**
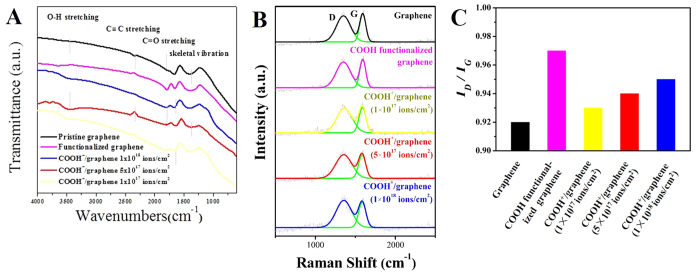
FTIR spectrum (**A**). Raman spectrum (**B**). *I*_*D*_/*I*_*G*_ spectrum depends on Raman (**C**).

As shown in Fig. 3A, the FTIR spectra of graphene show dominant peaks corresponding to skeletal vibration and C≡C stretching vibration^1^,^23^. And the COOH^+^/graphene and COOH functionalized graphene show the similar FTIR spectra. The broad absorption at 1750 cm^−1^ suggested the existence of C=O stretching vibration, confirming the formation of -COOH on the COOH^+^/graphene and COOH functionalized graphene^2^,^24^. In addition, the COOH^+^/graphene causes the appearance of absorption band centered at 3400 cm^−1^, which is attributed to the O-H stretching bands of hydroxy and carboxylic moieties. These results indicate that the COOH group was successfully introduced into COOH^+^/graphene and COOH functionalized graphene.

The Raman spectra of graphene, COOH^+^/graphene, and COOH functionalized graphene show the D-band at 1340 cm^−1^ and the G-band at 1580 cm^−1^ (Fig. 3B)^25^. The G band is assigned to the first order scattering of the E_2g_ phonon from *sp*^2^ carbon atoms. And the D band is attributed to a breathing mode of κ-point photons of A_1g_ symmetry which is related to the local *sp*^3^ disorder band formation especially the ones located at the edges of graphite sheets^2^. The intensity ratio (*I*_*D*_/*I*_*G*_) of the graphene increased from 0.92 to 0.97 after surface modified as shown in Fig. 3C. The high ratios of *I*_*D*_/*I*_*G*_ indicate the increased disorder in carbon materials^26^. Therefore, more defects are caused by the introduction of COOH. Different from FTIR, the Raman spectra are sensitive to the framework vibration of graphene (such as C-C) instead of the polar groups (such as C-O)^27^. As a result, after the COOH is introduced into graphene by the chemical and physical methods, both samples show similar *I*_*D*_/*I*_*G*_.

**Figure 4:**
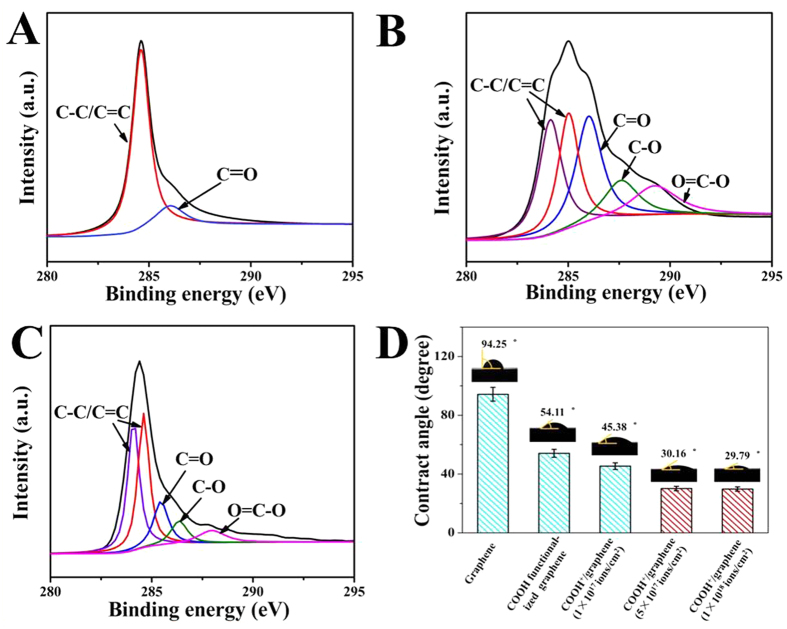
XPS C1s spectra of graphene (**A**), COOH functionalized graphene (**B**) COOH^+^/graphene (**C**), CA image of samples (**D**).

The XPS elemental analysis and peak fitting results are shown in Fig. 4(A–C). As shown in Fig. 4A, the C1s peak of graphene at ~285 eV is fitted into two peaks located at 285.7 and 284.6 eV, which match well with the C=O and C-C (*sp*^2^) groups, respectively^28^,^29^. In Fig. 4B and C, C1s XPS spectra of COOH functionalized graphene and COOH^+^/graphene show five peaks ascribed to C=C, C-C, C=O, C-O, O=C-O^26^,^28^,^29^,^30^,^31^, which means that COOH functional groups are successfully introduced into graphene *via* ion implantation and chemical method. These results are consistent with FTIR. On the other hand, the COOH functionalized graphene obviously shows a higher proportion of oxygen containing functional groups than COOH^+^/graphene. Based on the XPS results, the atomic percentage of oxygen in graphene, COOH functionalized graphene and COOH^+^/graphene with three inject doses (1 × 10^17^, 5 × 10^17^, 1 × 10^18^ ions/cm^2^) are 6.97 atm.%, 16.13 atm.%, 10.73 atm.%, 10.77 atm.% and 11 atm.%, respectively. Compared with COOH^+^/graphene, the COOH functionalized graphene contains more oxygen containing functional groups.

The graphene hydrophilicity was evaluated by contact angle method^26^. The inset images of Fig. 4D show that the contact angles images of graphene, COOH functionalized graphene and COOH^+^/graphene with three inject doses (1 × 10^17^, 5 × 10^17^, 1 × 10^18^ ions/cm^2^). And the bar chart (Fig. 4D) shows that the contact angles of these materials are 94.25 ± 2°, 54.11 ± 2°, 45.38 ± 2°, 30.16 ± 2° and 29.79 ± 2°, respectively. This illustrates that the existence of COOH effectively improves the hydrophilicity of graphene. Interestingly, COOH^+^/graphene has a smaller amount of oxygen, but its hydrophilicity is higher than COOH functionalized graphene. It might be because ion implantation only modifies the surface characteristics of graphene. Thus, a large number of COOH^+^ ions gather at the surface of COOH^+^/graphene with higher hydrophilicity.

### *In vitro* cytocompatibility studies

**Figure 5:**
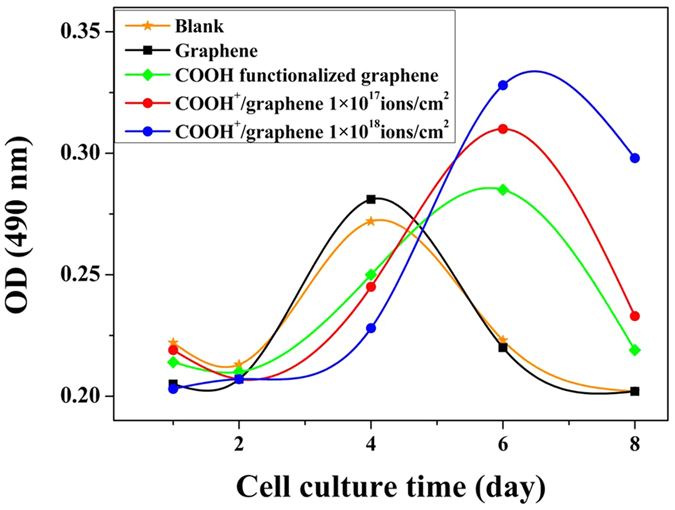
L929 fibroblast cell OD on the surface of graphene, COOH^+^/graphene, and COOH functionalized graphene.

L929 cells were used to estimate the cytocompatibility of samples by MTT assay *in vitro*. In Fig. 5, the numbers of living cells were recorded by optical density (OD) at 1d, 2d, 4d, 6d, 8d after they were seeded on the surface of graphene, COOH functionalized graphene, COOH^+^/graphene and culture plate (blank). As shown in Fig. 5, L-929 cells grow well on all the samples during a culture course. Differing from blank and graphene, the cells numbers of COOH functionalized graphene and COOH^+^/graphene show the maximum at 6d. And the proliferation of cells on COOH^+^/graphene is obviously enhanced compared with COOH functionalized graphene. Moreover, the cells numbers of COOH^+^/graphene increase with the dose of ion implantation. Compared with blank (24-well culture plate), the maximum cells of graphene and COOH functionalized graphene don’t significantly increase. But COOH^+^ ions implantation obviously improves the cytocompatibility of graphene.

**Figure 6:**
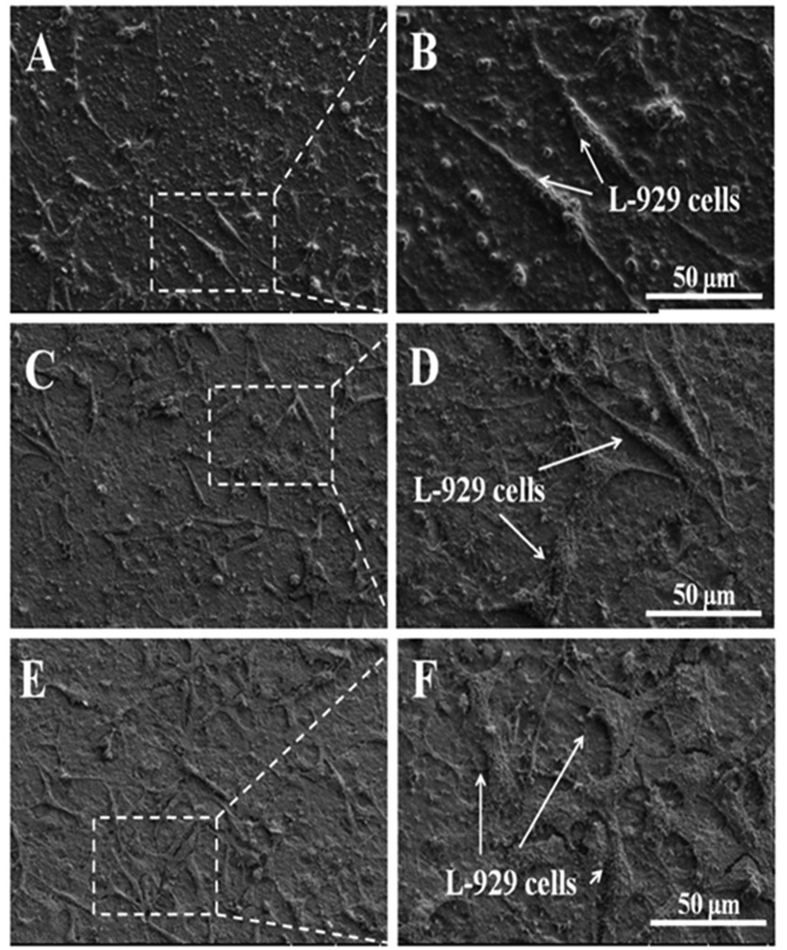
SEM images of L-929 fibroblast cells on graphene (**A,B**), COOH functionalized graphene (**C,D**) and COOH^+^/graphene (**E,F**).

To further clarify the samples’ cytocompatibility, the morphologies of cells seeded on graphene, COOH functionalized graphene and COOH^+^/graphene for 48 h were observed by SEM, as shown in Fig. 6. It indicates that the L-929 cells cultured on the COOH modified graphene show higher density than those on graphene (Fig. 6A,C,E). Especially, the surface of COOH^+^/graphene exhibits the largest ammout of L929 cells, and its cells are spread well with longer processus pseudopodia (Fig. 6F) than the COOH functionalized graphene (Fig. 6D) and graphene (Fig. 6B). The cells on graphene exhibit slender morphology, probably due to the poor adhesion of cells (Fig. 6B). As a result, it indicates that COOH^+^/graphene show the best effect to promote cells adhesion and proliferation.

## Discussion

First of all, the results of FT-IR and XPS verify that the COOH is successfully introduced into COOH^+^/graphene and COOH functionalized graphene (Figs 3 and 4). The COOH functionalized graphene holds more oxygen containing functional groups compared with COOH^+^/graphene, which is proved by Raman and XPS results (Figs 3B and 4B,C). More defects in carbon materials have been identified by Raman with the high ratios of *I*_*D*_/*I*_*G*_. Higher rates of C=O, C-O and O=C-O in COOH functionalized graphene were observed than those in COOH^+^/graphene, as shown in Fig. 4B,C. Generally, the more oxygen containing functional groups existed in materials’ surfaces with better hydrophilic property. However, the COOH^+^/graphene shows better hydrophilicity than COOH functional groups (Fig. 4D). It is because the carboxyl ion implantation does not affect the morphological microstructures of the graphene, as shown in Fig. 2, which means that the COOH^+^ ions mainly exist on the surfaces of COOH^+^/graphene. This illustrates that some COOH^+^ ions are affixed to graphene’s surfaces through electrostatic adsorption during the ion implantation process. One can see that the morphology of COOH functionalized graphene was completely changed after chemical functionalization. The graphene layers throughout oxidization process can be clearly identified as ‘building blocks’ and obvious aggregation, as shown in SEM and TEM images (Fig. 2G–I). It means the COOH functional groups are bonded to carbon atom of graphene *via* the chemical method.

According to the cytocompatibility study (Figs 5 and 6), COOH^+^/graphene shows the best cell adhesion and growth among three materials because COOH^+^/graphene surface holds more oxygen-containing functional groups compared with graphene, which is verified by FTIR, Raman and XPS analyses (Figs 3 and 4). And the oxygen-containing functional groups can make a significant contribution to improve the cell compatibility of materials. *In vitro* cytocompatibility study, the proliferation of L-929 cells on both COOH^+^/graphene and COOH functionalized graphene is enhanced compared with graphene after 5 days, as shown in Fig. 5. And it can be observed that the L-929 cells cultured on modified graphene showed higher density than that of graphene. SEM observation also showed that some L-929 cells adhered on graphene were nearly detached from the culture plates, and displayed morphological changes after 48 h, as shown in Fig. 6A,B. As a result, oxygen containing functional groups in COOH^+^/graphene and COOH functionalized graphene play an important impact on the biocompatibility.

In addition, compared with COOH functionalized graphene, the COOH^+^/graphene exhibits better performance in cytocompatibility. That is because the abundance of COOH^+^ ions existed on the surface of samples, which plays the important impact on the biocompatibility. The introduction of COOH^+^ ions does improve the hydrophilic property of graphene, moreover, it is conducive to negative charged cells adhesion, proliferation and viability. Besides, COOH^+^ ions are combined with the plasma proteins, which are belonging to the term of cell adhesion, by hydrogen bonds. This further enforces the attachment of cell on COOH^+^/graphene. Indeed, COOH^+^/graphene exhibited the highest cell-adhesion strength, cell viability, cell proliferation and cell stretching in MTT assay and SEM observation. The existence of COOH^+^ on the surface of graphene is the main reason to enhance cytocompatibility because the ion implantation technology could get more functional groups on the material surface than chemical functionalization.

With the increase of the implanted dosage of COOH^+^ ions, COOH^+^/graphene shows better hydrophobic by contact angle testing (Fig. 4D), which is beneficial to cell compatibility and growth. Therefore, the study demonstrated the good adhesion of COOH^+^/graphene with 1 × 10^18^ ions/cm^2^.

## Conclusions

Both COOH functionalized graphene and COOH^+^/graphene were successfully obtained by chemical functionalization and physical ion implantation, respectively. The results showed that carboxyl ion implantation did not affect the morphological microstructures of the graphene. More importantly, COOH^+^/graphene exhibited higher cell-adhesion strength, cell viability, cell proliferation and cell stretching. No obvious toxicity was observed on COOH functionalized graphene and COOH^+^/graphene. The existence of O-containing functional groups on the surface of samples might be the main reason of enhanced cytocompatibility. Ion implantation technology could get more functional groups on the material surface than chemical functionalization. Therefore, this study demonstrated the best cytocompatibility of COOH^+^/graphene with 1 × 10^18^ ions/cm^2^ as promising and effective biomedical material for biomaterial application.

## Methods

### Synthesis of graphene, COOH^+^/graphene and COOH functionalized graphene

Graphene was prepared from natural graphite *via* modified Hummers’ method, as we previously reported^32^. Then it was first dispersed in 1-Methy-2-Pyrrolidinone (NMP) solution by ultrasonication for 6 h^1^. The suspension was then sprayed on clean cycloidal polymethylmethacrylate (PMMA) substrate as a graphene support, followed by heating at 250 °C in a quartz tube at Ar atmosphere for 3 h to completely evaporate residual NMP^33^. The substrate coated with graphene (0.5 mg/cm^2^) was then removed from the tube center before being cooled from 250 °C to room temperature.

A Kaufmann-100 implanter was performed to obtain COOH^+^/graphene. The COOH^+^ was obtained from formic acid which was heated and evaporated into ion source^34^. The obtained COOH^+^ ions were accelerated at a high voltage onto the graphene samples located at the vacuum chamber. The implantation was done at accelerating power of 30 KeV, the beam current density of 119 μA/cm^2^, and the pressure of 1.5 × 10^−2^ Pa. The COOH^+^/graphene with three doses, 1 × 10^17^ ions/cm^2^, 5 × 10^17^ ions/cm^2^, and 1 × 10^18^ ions/cm^2^, were produced by adjusting the treatment time. The detail process of sample preparation can be found in our previous work^35^.

For comparison, graphene was also functionalized *via* a chemical method. In the first step, the as-prepared graphene was added into liquid mixture (3% conc. nitric acid + 3% conc. sulfuric acid + 79% water), and heated at 120 °C for 12 h. Then, the sample was repeatedly washed with distilled water and centrifuged to remove traces of acid. Finally, the graphene was functionalized with -COOH groups.

### SEM, TEM, XPS, Contact angle analyses

The morphologies of graphene, COOH^+^/graphene and COOH functionalized graphene were observed by field emission scanning electron microscopy (FE-SEM, Hitachi SU 8010) and high resolution transmission electron microscopy (HR-TEM, JEOL JEM-2100). The Fourier transform infrared spectroscopy (FTIR, MAGNA-560) was employed to characterize the surface functional groups of samples. Raman spectroscopy (Renishaw Invia Plus laser Raman spectrometer, Renishaw) and X-ray photoelectron spectroscopy (XPS, Kratos Axis Ultra Al) were used to identify the functional groups on the surface of materials. The water contact angles (CA) of samples were recorded using the CAM KSV021733 optical contact-angle inclinometer (Nunc, Finland).

### Cell viability and morphology

Cytocompatibility is an essential requirement for nanomaterials to be employed in biological systems, which was recommended by the International Standard Organization (ISO) as biocompatibility test model *in vitro*^1^,^36^. Mouse fibroblast cell (L-929) is considered to be an idea model cell line to study cell biocompatibility^37^, which is commonly used to evaluate cytotoxicity of potential substrates for cell growth^10^,^11^. In order to investigate the cytocompatibility of graphene, COOH^+^/graphene, and COOH functionalized graphene *in vitro*, L-929 cell line was chosen as the target cells to evaluate the cell viability and proliferation by [3-(4, 5-dimethylthiazol-2-yl)-2, 5-diphenyltetrazolium bromide] (MTT) assay^1^,^33^. The cells were cultured in a humidified 5% CO_2_/95% air incubator at 37 °C where the medium was replaced every 2 days. The cell culture medium (Roswell Park Memorial Institute-1640) was supplemented with 10% of fetal bovine serum (FBS, Gibco) and 100 units/ml streptomycin/penicillin^12^. The samples were sterilized and placed in 96-well cell culture plates. After an incubation period of 48 h, the cells (the inoculum density of L929 cells were 1 × 10^4^ cells/ml) were seeded onto each sample and allowed to culture from day 1 to day 8 at 37 °C in a humidified 5% CO_2_ incubator^29^,^38^. Except from experiment group, the contents in the remaining group were added into medium without samples taken as the blank (control group)^9^,^12^,^29^,^36^. The samples were incubated with cells in 96-well cell culture plates. After incubation for 24, 48, 96, 144 and 192 h, cells were added with a total of 20 μL MTT solution (M5655, Sigma, 5 mg/mL in PBS solution) and incubated for 3 h. 150 μL dimethyl sulfoxide (DMSO) was added to each sample and incubated for an additional 0.5 h at 37 °C^1^,^9^. After incubation, culture supernatants were aspirated, and purple insoluble MTT product formazan was dissolved in DMSO solution^1^. The absorbency which could reflect the survival rate of cells was measured using the Biotek ELx808 plate reader at 490 nm according to the MTT assay^26^. The blank absorbance (medium without cells) was subtracted from each value. All the experiments were conducted in triplicate^12^. Cell morphology and stretching on graphene, COOH^+^/graphene, and functionalized graphene were observed under the scanning electron microscopy (SEM, FEI QUANTA 200) at 48 h post exposure^9^.

## Additional Information

**How to cite this article**: Zhao, M.-l. *et al*. Enhancement of interaction of L-929 cells with functionalized graphene *via* COOH^+^ ion implantation *vs.* chemical method. *Sci. Rep.* **6**, 37112; doi: 10.1038/srep37112 (2016).

**Publisher’s note:** Springer Nature remains neutral with regard to jurisdictional claims in published maps and institutional affiliations.

## Figures and Tables

**Figure 1:**
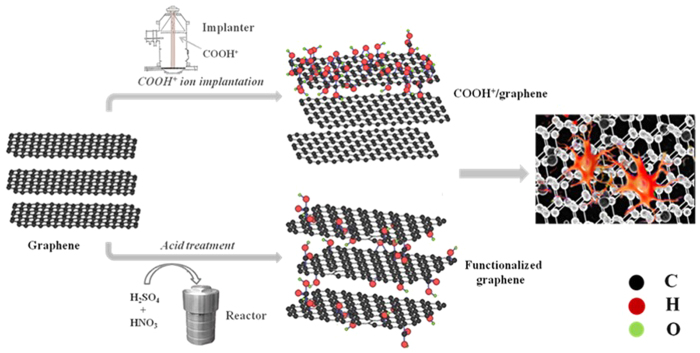
Schematic illustration of the formation of COOH^+^/graphene and COOH functionalized graphene. Then study their biological effects with L-929 fibroblast cells.
